# Supplementary material for: Effect of helical kink in antimicrobial peptides on membrane pore formation
Source: eLife. 2020 Mar 13;9:e47946. doi: 10.7554/eLife.47946 (PMC7069690; doi:10.7554/eLife.47946)
Supplement: Transparent reporting form [file elife-47946-transrepform.pdf]

eLIFE
1st Floor
24 Hills Road
Cambridge CB2 1JP, UK
P 01223 855340
W elifesciences.org
T @elife

eLife's transparent reporting form

We encourage authors to provide detailed information *within their submission* to facilitate the interpretation and replication of experiments. Authors can upload supporting documentation to indicate the use of appropriate reporting guidelines for health-related research (see EQUATOR Network), life science research (see the BioSharing Information Resource), or the ARRIVE guidelines for reporting work involving animal research. Where applicable, authors should refer to any relevant reporting standards documents in this form.

If you have any questions, please consult our Journal Policies and/or contact us: editorial@elifesciences.org.

Sample-size estimation

- You should state whether an appropriate sample size was computed when the study was being designed
- You should state the statistical method of sample size computation and any required assumptions
- If no explicit power analysis was used, you should describe how you decided what sample (replicate) size (number) to use

Please outline where this information can be found within the submission (e.g., sections or figure legends), or explain why this information doesn't apply to your submission:

Our study mainly involves computer simulations, where the sample-size estimation is not applicable. The details can be found in the Methods section.

Replicates

- You should report how often each experiment was performed
- You should include a definition of biological versus technical replication
- The data obtained should be provided and sufficient information should be provided to indicate the number of independent biological and/or technical replicates
- If you encountered any outliers, you should describe how these were handled
- Criteria for exclusion/inclusion of data should be clearly stated
- High-throughput sequence data should be uploaded before submission, with a private link for reviewers provided (these are available from both GEO and ArrayExpress)

Please outline where this information can be found within the submission (e.g., sections or figure legends), or explain why this information doesn't apply to your submission:

All simulations were performed as single trajectories. However, the data were averaged over the time and we provide standard deviation when applicable. In free energy calculations, the simulations run until the convergence limit is met and the details about it are in the Methods section. For Martini coarse-grained simulations, we used number of different initial configurations. Decision made on a particular initial condition was based on the short NVT simulation (t=3 μs) where a pre-formed membrane pore was fixed, so the peptides were capable to adopt a distinct orientation within a pre-formed pore. In case of ambiguous peptide orientations, more initial configurations (up to three distinct configurations per a simulated system) were prepared. Specifically, simulations with buforin II peptides were initiated by using two distinct configurations (Figure 6A), LL-37 peptides by using single configuration (Figure 6B), delta-lysin peptides by using single configuration (Figure 6C), candidalysin peptides by using three distinct configurations (Figure 6D), and magainin II peptides by using two distinct configurations (Figure 6E), respectively.

Fluorescence leakage assay was performed three times for each studied peptide. The replicates were performed with independetly-prepared fresh batch of lipid vesicles. All experimental measurements are presented in the results.

- Statistical analysis methods should be described and justified
- Raw data should be presented in figures whenever informative to do so (typically when N per group is less than 10)
- For each experiment, you should identify the statistical tests used, exact values of N, definitions of center, methods of multiple test correction, and dispersion and precision measures (e.g., mean, median, SD, SEM, confidence intervals; and, for the major substantive results, a measure of effect size (e.g., Pearson's r, Cohen's d)
- Report exact p-values wherever possible alongside the summary statistics and 95% confidence intervals. These should be reported for all key questions and not only when the p-value is less than 0.05.

Please outline where this information can be found within the submission (e.g., sections or figure legends), or explain why this information doesn't apply to your submission:

Standard averaging was used for experiments and simulations. Water density analysis was performed on last 15 μs simulations. For free energy calculation no statistical analysis was performed and the error is estimated from the roughness of the profiles. All simulation and analysis methods are described in the paper.

(For large datasets, or papers with a very large number of statistical tests, you may upload a single table file with tests, Ns, etc., with reference to sections in the manuscript.)

Group allocation

- Indicate how samples were allocated into experimental groups (in the case of clinical studies, please specify allocation to treatment method); if randomization was used, please also state if restricted randomization was applied
- Indicate if masking was used during group allocation, data collection and/or data analysis

eLife Sciences Publications, Ltd is a limited liability non-profit non-stock corporation incorporated in the State of Delaware, USA, with company number 5030732, and is registered in the UK with company number FC030576 and branch number BR015634 at the address 1st Floor, 24 Hills Road, Cambridge CB2 1JP | August 2014

Please outline where this information can be found within the submission (e.g., sections or figure legends), or explain why this information doesn't apply to your submission:

Group allocation is not applicable to the present study.

Additional data files ("source data")

- We encourage you to upload relevant additional data files, such as numerical data that are represented as a graph in a figure, or as a summary table
- Where provided, these should be in the most useful format, and they can be uploaded as "Source data" files linked to a main figure or table
- Include model definition files including the full list of parameters used
- Include code used for data analysis (e.g., R, MatLab)
- Avoid stating that data files are "available upon request"

Please indicate the figures or tables for which source data files have been provided:

The source data for the whole study have size of 1 TB, which makes it difficult to upload this data to a standard repository. Nevertheless, the trajectories are stored on our local storage and is available upon request. Source files for Figures 1-6 are provided including both the data files for plots and configuration files for snapshots.

The code used to obtain the data including simulation programs are accessible as part of freely available software cited in the manuscript. Simulation parameters and performed analysis of the data is clearly described in the Methods section of the manuscript to facilitate reproducibility of results.
